# The Influences of TSH Stimulation Level, Stimulated Tg Level and Tg/TSH Ratio on the Therapeutic Effect of ^131^I Treatment in DTC Patients

**DOI:** 10.3389/fendo.2021.601960

**Published:** 2021-06-11

**Authors:** Wei Zheng, Zhongying Rui, Xuan Wang, Ning Li, Jian Tan, Wei Liu

**Affiliations:** ^1^ Department of Nuclear Medicine, Tianjin Medical University General Hospital, Tianjin, China; ^2^ Department of Otolaryngology Head and Neck Surgery, Tianjin Fourth Central Hospital, Tianjin, China

**Keywords:** differentiated thyroid cancer, ^131^I treatment, TSH stimulation level, sTg, sTg/TSH ratio, therapeutic effect

## Abstract

**Purpose:**

To study the influences of pre-ablation TSH stimulation level, sTg and sTg/TSH ratio on the therapeutic effect of the first ^131^I treatment in DTCs.

**Methods:**

According to the thyroid stimulating hormone (TSH) levels (mU/l), all the 479 differentiated thyroid cancer (DTC) patients were divided into two groups: TSH < 30 and TSH ≥ 30. The TSH ≥ 30 group was divided into three subgroups: 30 ≤ TSH < 60, 60 ≤ TSH < 90 and TSH ≥ 90. The clinical features and the therapeutic effects of the first ^131^I treatment were analyzed. The cutoffs of stimulated thyroglobulin (sTg) and sTg/TSH ratio were calculated to predict the therapeutic effect of ^131^I treatment.

**Results:**

Among the three subgroups, the TSH ≥ 90 subgroup was younger and less likely to be associated with cervical lymph node metastasis (LNM). The postoperative levothyroxine (L-T_4_) dose in the 60 ≤ TSH < 90 subgroup was the lowest. Between the two groups, patients in the TSH < 30 group had higher postoperative L-T_4_ dose and longer thyroid hormone withdrawal (THW) time. The excellent response rates six months after the first ^131^I treatment among the three subgroups and between the two groups were not of statistical significance. The distribution of different TSH stimulation levels among each response group was similar. The cutoffs for the better therapeutic effect of the first ^131^I treatment in sTg and sTg/TSH were < 9.51 ng/ml and < 0.11, respectively. Both univariate and multivariate logistic regressions showed that cervical LNM, distant metastasis, higher sTg and higher sTg/TSH ratio predicted poorer therapeutic effect.

**Conclusions:**

There was no significant influence of TSH stimulation levels before the first ^131^I treatment on the therapeutic effect of DTC. The sTg/TSH ratio can be considered as another predictor of ^131^I therapeutic effect.

## Introduction

Thyroid cancer is the most common endocrine malignancy. Differentiated thyroid cancer (DTC) with two subtypes, papillary thyroid cancer (PTC) and follicular thyroid cancer (FTC), is the most frequent subtype of thyroid cancer. For DTC patients, under the concept of multidisciplinary and comprehensive treatment, selective postoperative ^131^I treatment may be considered to improve prognosis (1). The 2015 American Thyroid Association (ATA) guidelines for the management of adult patients with thyroid nodules and DTC strongly recommend, with moderate quality of evidence, that thyroid stimulating hormone (TSH) stimulation level ≥ 30 mU/l over a three to four week period of levothyroxine (L-T_4_) withdrawal should be performed for those DTC patients in whom ^131^I treatment is planned (2). Elevated TSH stimulation level is ensured to maximize ^131^I uptake in thyroid remnants and presumably malignant cells during postoperative ablation. Indeed, this recommendation is based solely on an old paper published in 1977 (3). It is controversial that some clinicians believe that DTC patients with higher TSH stimulation levels will achieve a better clinical response than patients with lower TSH levels (4, 5). In contrast, other researchers demonstrated that DTC patients achieving TSH stimulation levels < 30 mU/l or thyroid hormone withdrawal (THW) less than two weeks before ^131^I treatment did not show a significant difference in treatment effect in comparison with those whose TSH stimulation levels were ≥ 30 mU/l or THW lasting three to four weeks (6, 7).

Thyroglobulin (Tg) is a well-known marker in the follow-up of DTC patients after thyroidectomy and ^131^I treatment for persistent disease, distant metastasis, or disease recurrence (8, 9). The stimulated Tg (sTg) before ^131^I treatment is a good predictor of successful ablation (10), which can be influuenced by tumor staging and TSH levels as well as other factors.

In this study, we aimed to determine whether TSH stimulation levels influence the therapeutic effect of the first ^131^I treatment in DTC patients and to use TSH to correct the predictive value of the sTg to see whether sTg/TSH ratio can be used as another predictor of ^131^I therapeutic effect.

## Patients and Methods

We reviewed the clinical records of patients with DTC followed at the Nuclear Medicine Department of Tianjin Medical University General Hospital between January 2014 and December 2018. A total of 479 DTC patients were enrolled in this study. All of them received total thyroidectomy and underwent postoperative ^131^I treatment at least once in our department. All patients were instructed to follow a low iodine diet one month before, during, and one month after ^131^I treatment. Between surgery and ^131^I ablation, L-T_4_ treatment was withheld. With respect to the ^131^I dose, 100 mCi was recommended for regular patients, 100–150 mCi for patients with cervical lymph node metastasis (LNM) and/or extrathyroid extension (ETE), and 150–200 mCi is generally recommended if distant metastasis exists. According to the 2015 ATA risk stratification, all of the above 479 patients were intermediate-high risk patients, and the appropriate TSH suppression therapy was routinely given to all patients (TSH < 0.1 mU/l). Prior to ^131^I treatment, TSH stimulation levels were measured. Patients with large thyroid remnants as a treatable cause for low TSH levels or using recombinant human TSH (rhTSH) were excluded.

All patient charts were reviewed and analyzed retrospectively. The patients were divided into two groups according to TSH stimulation levels (mU/l): TSH < 30 and TSH ≥ 30. We retrospectively analyzed clinical features and therapeutic effects after the first ^131^I treatment between the two groups. Thereafter, we divided the TSH ≥ 30 patients into three subgroups: 30 ≤ TSH < 60, 60 ≤ TSH < 90 and TSH ≥ 90. A further study of clinical features and therapeutic effects after the first ^131^I treatment was analyzed. We then assessed the sTg level and sTg/TSH ratio before the first ^131^I treatment as possible predictors of ^131^I therapeutic effect.

Follow-up information was obtained from a database containing patients’ medical records. We collected clinical and laboratory assessments six months after the first ^131^I treatment. According to the 2015 ATA guidelines (2), we divided the patients into ①excellent response (ER): negative imaging and either suppressed Tg < 0.2 ng/mL or sTg < 1 ng/ml; ②indeterminate response (IDR): nonspecific findings on imaging studies, faint uptake in thyroid bed on ^131^I scanning, nonstimulated Tg detectable but < 1 ng/ml, sTg detectable but < 10 ng/ml, or anti-Tg antibody (TGAb) stable or declining in the absence of structural or functional disease; ③biochemical incomplete response (BIR): negative imaging and suppressed Tg ≥1 ng/ml or sTg ≥10 ng/ml or rising TGAb levels, and ④structural incomplete response (SIR): structural or functional evidence of disease, with any Tg level, with or without TGAb. ER and IDR predicted good therapeutic effects, and BIR and SIR predicted poor therapeutic effects.

Data and statistical tests were carried out using SPSS (version 22.0; SPSS Inc., Chicago, Illinois, USA). Descriptive statistics were used to summarize the data. Quantitative data were expressed as mean ± SD. Differences in categorical values between groups were evaluated using the chi-squared test. Differences in continuous variables between groups were evaluated using the t test or ANOVA if normally distributed or the Mann-Whitney or Kruskal-Wallis test, if not. The Spearman correlation test was used to test the relationships between sTg, TSH and therapeutic effects, respectively. Values with maximum sensitivity and specificity were selected to define cutoff values. Univariate and multivariate logistic regression were performed to assess the predictors of successful ablation. Univariate analysis was first performed by entering the variables concerned into the regression analysis. Any variable that showed a *p* value of < 0.2 in the univariate analysis was then included in the multivariate regression analysis, using forward selection procedures. For the purpose of multivariate analysis, TSH levels were entered as a continuous variable. A *p* value <0.05 was considered statistically significant.

## Results

The study included 479 DTC patients, 31 patients in the TSH < 30 group and 448 patients in the TSH ≥ 30 group. The number of patients in the three subgroups of the TSH ≥ 30 group were 125 (30 ≤ TSH < 60), 127 (60 ≤ TSH < 90) and 196 (TSH ≥ 90), respectively.

First, the characteristics of the three subgroups of the TSH ≥ 30 group were summarized in **Table 1**. The three subgroups were similar in composition. No statistically significant differences were found in sex, body mass index (BMI), histology, background of benign thyroid diseases (BTDs, including Hashimoto’s thyroiditis, Graves’ disease and goiters), sTg, THW, ^131^I dose and most tumor associated features among the three subgroups (all p > 0.05). Patients in the TSH ≥ 90 subgroup were younger than the other two subgroups (39.63 ± 12.38 vs. 49.28 ± 11.48 and 45.77 ± 11.39, p = 0.000). There were also more patients in the TSH ≥ 90 subgroup without cervical LNM (11.22% vs. 8.00% and 6.30%, p = 0.018). Regarding the postoperative L-T_4_ dose (μg/d), patients in the 60 ≤ TSH < 90 subgroup took the lowest dose of L-T_4_ among the three subgroups (82.68 ± 29.31 vs. 93.10 ± 26.58 and 87.42 ± 35.29, p = 0.045).

**Table 1:** Characteristics of patients with TSH stimulation levels (mU/l) 30 ≤ TSH < 60, 60 ≤ TSH < 90 and TSH ≥ 90 before the ^131^I treatment.

	30 ≤ TSH < 60	60 ≤ TSH < 90	TSH ≥ 90	*p* value
Number of patients	125	127	196	
Median TSH	46.48 (30.05-59.95)	74.57 (60.32-89.45)	104.65 (90.23->150)	
Average TSH	45.57 ± 7.89	74.08 ± 8.51	117.75 ± 22.59	
Age(years)	49.28 ± 11.48	45.77 ± 11.39	39.63 ± 12.38	0.000*
Sex				
Male	43 (34.40)	36 (28.35)	73 (37.24)	0.274
Female	82 (65.60)	91 (71.65)	123 (62.76)	
BMI	25.37 ± 4.19	26.05 ± 4.35	25.55 ± 4.47	0.506
Histology				
PTC	121 (96.80)	124 (97.64)	195 (99.49)	0.450
FTC	4 (3.20)	3 (2.36)	1 (0.51)	
Tumor size				
T1	50 (40.00)	49 (38.58)	81 (41.33)	0.964
T2	11 (8.80)	10 (7.87)	11 (5.61)	
T3	39 (31.20)	46 (36.22)	73 (37.24)	
T4	25 (20.00)	22 (17.32)	31 (15.82)	
Lymph node status				
N0	10 (8.00)	8 (6.30)	22 (11.22)	0.018*
N1	115 (92.00)	119 (93.70)	174 (88.78)	
Distant metastasis				
M0	96 (76.80)	103 (81.10)	160 (81.63)	0.638
M1	29 (23.20)	24 (18.90)	36 (18.37)	
ETE				
No	35 (28.00)	66 (51.97)	95 (48.47)	0.120
Yes	90 (72.00)	61 (48.03)	101 (51.53)	
Background of BTDs				
No	76 (60.80)	91 (71.65)	172 (87.76)	0.753
Yes	49 (39.20)	36 (28.35)	24 (12.24)	
sTg (ng/mL)	32.99 ± 76.21	32.86 ± 82.85	26.02 ± 54.52	0.594
L-T4 (μg/d)	93.10 ± 26.58	82.68 ± 29.31	87.42 ± 35.29	0.045*
THW (d)	21.99 ± 7.11	21.58 ± 5.95	22.74 ± 5.10	0.275
^131^I dose (mCi)	116.92 ± 33.38	115.33 ± 24.90	116.18 ± 27.50	0.911

Data are presented as n (%) or as the mean ± SD.

BMI, body mass index; BTD, benign thyroid disease; ETE, extrathyroid extension; FTC, follicular thyroid cancer; L-T4, levothyroxine; PTC, papillary thyroid cancer; sTg, stimulated thyroglobulin; THW, thyroid hormone withdrawal; TSH, thyroid stimulating hormone.

*p < 0.05.

**Figure 1:**
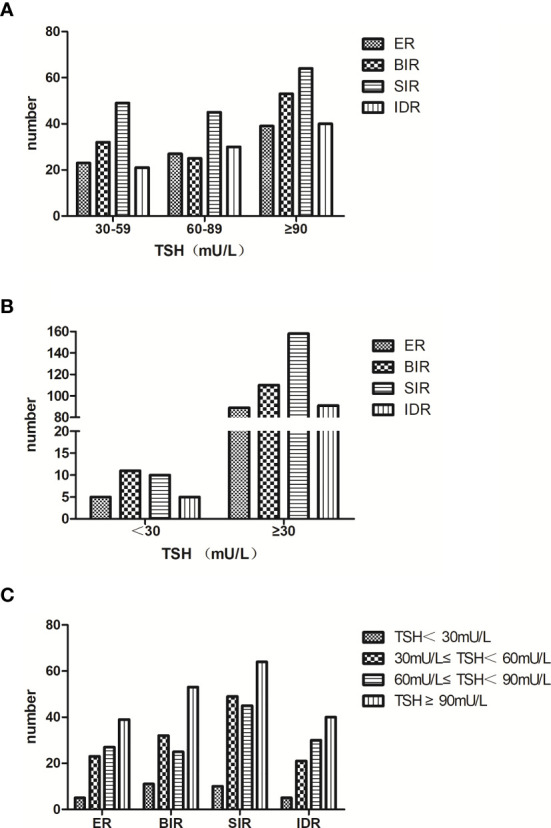
**(A)** we divided the TSH ≥ 30 patients into three subgroups: 30 ≤ TSH < 60, 60 ≤ TSH < 90 and TSH ≥ 90. The response rate six months after the first ^131^I treatment among the three subgroups was of no statistical significance (*p* = 0.657). **(B)** we merged the three subgroups into the TSH ≥ 30 group. Compared with the TSH < 30 group, the response rate six months after the first ^131^I treatment was still similar (*p* = 0.532). **(C)** the distribution of patients with different TSH stimulation levels in each response group was similar (*p* = 0.703).

Subsequently, analysis of therapeutic effects after the first ^131^I treatment was carried out in these TSH ≥ 30 patients. Patients showing good therapeutic effects (ER + IDR) among the three subgroups were 35.20%, 44.88% and 40.31%, respectively, which showed that the response rate six months after the first ^131^I treatment among the three subgroups was not of statistical significance (*p* = 0.657) (**Figure 1A**).

We then merged the three subgroups into the TSH ≥ 30 group. The characteristics of the TSH < 30 group and the TSH ≥ 30 group were summarized in **Table 2**. Like the three subgroups above, these two groups were still similar in composition, and no statistically significant differences were found in age, sex, BMI, histology, background of BTDs, sTg, ^131^I dose and all tumor associated features (all p > 0.05). Statistical significances were only observed for L-T_4_ dose and THW. In the TSH < 30 group, the postoperative L-T_4_ dose (μg/d) was higher (106.47 ± 34.82 vs. 87.79 ± 3 1.43, p = 0.002) and THW (d) was longer (26.21 ± 8.13 vs. 22.19 ± 6.00, p = 0.016).

**Table 2:** Characteristics of patients with TSH stimulation levels (mU/l) < 30 and ≥ 30 before the ^131^I treatment.

	TSH < 30	TSH ≥ 30	*p* value
Number of patients	31	448	
Median TSH	20.17 (0.02-29.55)	82.08 (30.05->150)	
Average TSH	17.51 ± 9.37	85.28 ± 34.64	
Age(years)	46.67 ± 11.88	44.06 ± 12.53	0.269
Sex			
Male	9 (29.03)	152 (33.93)	0.696
Female	22 (70.97)	296 (66.07)	
BMI	29.96 ± 25.86	25.64 ± 4.35	0.404
Histology			
PTC	29 (93.55)	440 (98.21)	0.205
FTC	2 (6.45)	8 (1.79)	
Tumor size			
T1	13 (41.94)	180 (40.18)	0.144
T2	4 (12.90)	32 (7.14)	
T3	5 (16.13)	158 (32.27)	
T4	9 (29.03)	78 (17.41)	
Lymph node status			
N0	3 (9.68)	40 (8.93)	0.756
N1	28 (90.32)	408 (91.07)	
Distant metastasis			
M0	26 (83.87)	359 (80.13)	0.654
M1	5 (16.13)	89 (19.87)	
ETE			
No	13 (41.94)	252 (56.25)	0.526
Yes	18 (58.06)	196 (43.75)	
Backgrounds of BTDs			
No	6	339	0.511
Yes	25	109	
sTg (ng/mL)	43.95 ± 77.50	29.90 ± 69.64	0.290
L-T4 (μg/d)	106.47 ± 34.82	87.79 ± 31.43	0.002*
THW (days)	26.21 ± 8.13	22.19 ± 6.00	0.016*
^131^I dose (mCi)	120.00 ± 29.36	116.14 ± 28.53	0.475

Data are presented as n (%) or as the mean ± SD.

BMI, body mass index; BTD, benign thyroid disease; ETE, extrathyroid extension; FTC, follicular thyroid cancer; L-T4, levothyroxine; PTC, papillary thyroid cancer; sTg, stimulated thyroglobulin; THW, thyroid hormone withdrawal; TSH, thyroid stimulating hormone.

*p < 0.05.

There were 5 (16.13%) ER patients, 5 (16.13%) IDR patients, 11 (35.48%) BIR patients and 10 (32.26%) SIR patients in the TSH < 30 group, which were 89 (19.87%), 91 (20.31%), 110 (24.55%) and 158 (35.27%) in the TSH ≥ 30 group, respectively. The response rate six months after the first ^131^I treatment between the two groups was not statistical significance (*p* = 0.532) (**Figure 1B**).

The relationship between TSH stimulation levels and therapeutic effects after the first ^131^I treatment for all patients was analyzed. Our results showed that fewer patients in the TSH < 30 group obtained a good therapeutic effect (32.26% vs. 35.20%, 44.88% and 40.31%). However, the response rates of patients with varying TSH stimulation levels made no significant difference (*p* = 0.703). The distributions of patients with different TSH stimulation levels in each response group were similar(**Figure 1C**).

The relationships between TSH stimulation level, sTg level and therapeutic effect after the first ^131^I treatment were analyzed using Spearman’s correlation coefficient test. A negative correlation was found between sTg levels and ^131^I therapeutic effects (r = -0.133, *p* = 0.004). A lower sTg level was associated with a better therapeutic effect. A positive correlation was found between TSH stimulation levels and therapeutic effects, but there was still no significant difference between them (r = 0.004, *p* = 0.935) (**Table 3**).

**Table 3:** Relationship between TSH stimulation level, sTg level and treatment outcome.

Variables	TSH stimulation level	sTg level
TSH stimulation level	1	–
sTg level	–	1
Therapeutic effects [r (*p*)]	0.004 (0.935)	-0.133 (0.004*)

r, Spearman’s correlation coefficient; sTg, stimulated thyroglobulin; TSH, thyroid stimulating hormone. *p < 0.05.

**Figure 2:**
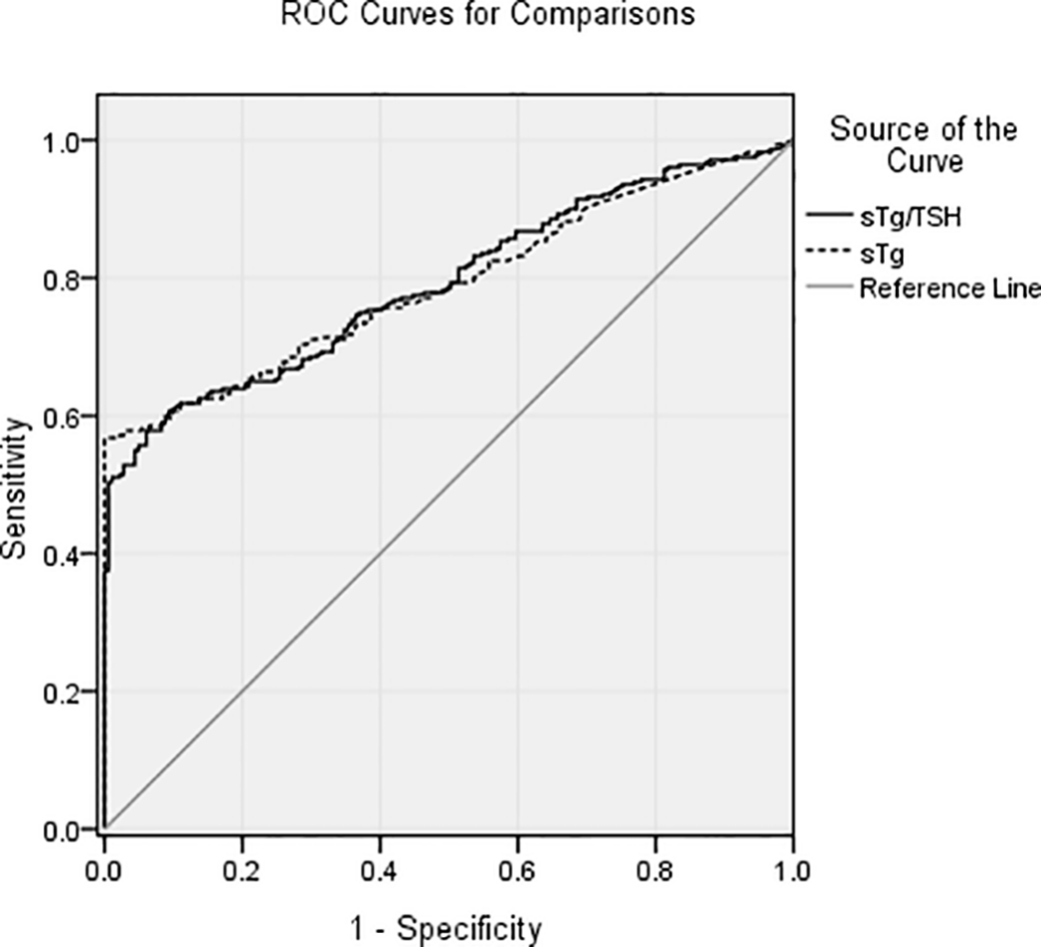
The cutoffs for sTg level and sTg/TSH ratio were 9.51 ng/ml (AUC: 0.790) and 0.11 (AUC: 0.792), respectively. These values had high specificity and moderate sensitivity to predict a better therapeutic effect of the first ^131^I treatment.

According to the ROC curve (**Figure 2**), the cutoffs for sTg level and sTg/TSH ratio were 9.51 ng/ml (area under the curve (AUC): 0.790) and 0.11 (AUC: 0.792), respectively. These values had high specificity and moderate sensitivity to predict a better therapeutic effect.

Univariate logistic regression found no significant association of therapeutic effects with age, sex, BMI, TSH stimulation level, tumor size, ETE, histology and background of BTDs. LNM, distant metastasis, sTg > 9.51 ng/mL, and sTg/TSH > 0.11 indicated poor therapeutic effects. Slight differences persisted in multivariate analysis that male, high BMI and ETE remained significantly associated with poor therapeutic effects except for LNM, distant metastasis and higher sTg, sTg/TSH ratio (**Table 4**).

**Table 4:** Univariate analysis and multivariate analysis of tumor characteristics and therapeutic effect.

Variables	Univariate analysis		Multivariate analysis	
	OR (95% CI)	*p* value	OR (95% CI)	*p* value
Age	0.988 (0.974-1.003)	0.125	0.982 (0.960-1.005)	0.122
Sex	0.762 (0.515-1.129)	0.175	2.229 (1.061-4.679)	0. 034*
BMI	0.971 (0.932-1.012)	0.159	0.920 (0. 858-0.986)	0. 018*
TSH	0.997 (0.992-1.002)	0.220		
Tumor size	1.476 (0.931-2.340)	0.098	0.962 (0.581-1.592)	0. 879
ETE	0.522 (0.265-1.029)	0.061	0. 481 (0.235-0.986)	0.046*
Histology	2.342 (0.481-11.396)	0.292		
BTDs	0.890 (0.427-1.855)	0.757		
LNM	2.166 (1.141-4.112)	0.018*	2.489 (1.265-4.898)	0.008*
Distant metastasis	3.154 (1.834-5.424)	0.000*	4.719 (2.472-9.008)	0.000*
sTg	1.214 (1.147-1.284)	0.000*	1.223 (1.144-1.308)	0.000*
sTg/TSH	3.091(2.144-4.455)	0.000*	3.508 (2.002-6.149)	0.000*

BMI, body mass index; BTD, benign thyroid disease; ETE, extrathyroid extension; LNM, lymph node metastasis; sTg, stimulated thyroglobulin; TSH, thyroid stimulating hormone.

*p < 0.05.

## Discussion

Thyroid cancer is the most common malignancy affecting any endocrine organ. Although its incidence has been on the increase, its mortality rate has been stable or even on the decline in different parts of the world (11). The mainstay of treatment for DTC is total or near-total thyroidectomy, followed in most cases by radioiodine ablation of remnant thyroid tissue/malignant thyroid tissue and thyroid hormone suppression therapy. It was first reported in a study more than 40 years ago that DTC patients who failed to produce TSH stimulation levels >30 mU/l over a postoperative THW period of 4 - 6 weeks showed much lower rates of successful ^131^I treatment (3). The threshold has been quoted in the international literature, including many guidelines, as the minimum level of TSH stimulation for ^131^I treatment (2, 12–15). The physiological rationale behind high level TSH stimulation before ^131^I treatment is well established as in response to TSH stimulation, thyrocytes upregulate the expression, transcription and activity of the sodium iodine symporter (NIS) (16–19). However, the studies mentioned above were published more than a decade ago.

In clinical practice, DTC patients scheduled for ^131^I treatment must achieve TSH stimulation levels ≥ 30 mU/l. Usually 3 - 4 weeks of THW are deemed necessary to reach these target TSH levels before ^131^I treatment. However, this arbitrary cut-off was derived long ago from the TSH level necessary for increased ^131^I uptake rather than the optimal level to achieve better prognosis after ^131^I treatment. Some researchers believed that a TSH of > 30 mU/l or THW over two weeks was not in itself required for successful ^131^I treatment (6, 7). On the contrary, others suggested that DTC patients might achieve a better response to ^131^I treatment after a stronger TSH stimulation level due to a probable higher ^131^I uptake (4, 5).

In our paper, we first studied patients whose TSH stimulation levels reached 30 mU/l. We divided these 448 patients into three subgroups: 30 ≤ TSH < 60, 60 ≤ TSH < 90 and TSH ≥ 90. We found that patients in the TSH ≥ 90 subgroup were younger and with less cervical LNM than the other two subgroups, which conformed to the clinical conditions and showed that youth was a protective factor of the cervical LNM. Whereas the patients in the 60 ≤ TSH < 90 subgroup took the lowest dose of L-T_4_ among the three subgroups, this indicated that the postoperative L-T_4_ dose was not the only key factor for the withdrawal time. Most of the remaining clinical features were similar among the three subgroups and there was no statistical significance on therapeutic effects six months after the first ^131^I treatment, which was inconsistent with the studies mentioned above (4, 5).

We then merged the three subgroups into the TSH ≥ 30 group. By comparison, the patients in the TSH < 30 group took more L-T_4_ and had longer withdrawal times. Comparing the rest characteristics and response rates six months after the first ^131^I treatment between the TSH < 30 group and the TSH ≥ 30 group, a similar trend was demonstrated. There was still no statistical significance for therapeutic effects six months after the first ^131^I treatment. The results obtained in our research coincided with Vrachimis’ (6). TSH stimulation levels before ^131^I treatment are not related to the prognosis. We found that after nearly four weeks of withdrawal time, the average TSH level in the TSH < 30 group was only 17.51 ± 9.37 mU/l, which was significantly lower than the average level in the TSH ≥ 30 group. Therefore, we believed that for a better therapeutic effect, the benefits of prompt ^131^I treatment were more important than longer withdrawal time.

Third, we analyzed the relationship between TSH stimulation level, sTg level and therapeutic effect using the Spearman correlation test. Only a lower sTg level predicted a better therapeutic effect and there was a positive correlation between TSH stimulation level and therapeutic effect without significant difference, which further illustrated that a higher TSH stimulation level was not in itself required for successful ^131^I treatment.

As sTg is actively released into circulation by DTC, its level in circulation following total or near-total thyroidectomy and ^131^I treatment is an established method of detecting recurrent or persistent disease and predicting the therapeutic effect of the treatment (20, 21). Measurement of sTg becomes an important parameter in the follow-up of DTC patients. A retrospective study revealed that a preablation sTg < 8.55 ug/l predicted disease remission after 18–24 months of ^131^I treatment with sensitivity of 88%, specificity of 72% (10). Another study found that patients with sTg > 18 ng/ml and sTg/TSH > 0.35 before ^131^I treatment were significantly associated with unsuccessful ablation (22), which was the first attempt to assess the association of sTg/TSH ratio with prognosis. In this study, we found that same as the sTg, sTg/TSH ratio had a strong association with the therapeutic effect of the first ^131^I treatment. This can help us in predicting the therapeutic effects of ^131^I treatment. The sTg < 9.51 ng/ml and sTg/TSH < 0.11 predicted a better therapeutic effect. These values had high specificity and moderate sensitivity.

However, a limitation of our study was its retrospective nature. We were able to retrieve data for only 31 patients whose TSH stimulation levels were lower than 30 mU/l. Another limitation of this study was that because we could not make sure all 31 patients maintained the TSH stimulation levels lower than 30 mU/l again before the second ^131^I treatment if necessary, we only analyzed the clinical features and the response after the first ^131^I treatment. The original study also showed that most patients who failed to achieve a TSH of > 30 mU/l at the time of postoperative ^131^I treatment did in fact achieve a TSH of > 30 mU/l before the second ^131^I treatment (3). This indicated that low TSH stimulation levels in that study were an indicator of insufficient surgery rather than insufficient physiological stimulus for ^131^I uptake.

In conclusion, there was no evidence that higher TSH stimulation levels predicted better therapeutic effects of the first ^131^I treatment. Even TSH < 30 mU/l will not influence the therapeutic effect. Meanwhile, before the first ^131^I treatment, sTg < 9.51 ng/mL and sTg/TSH < 0.11 predicted a better therapeutic effect. Our findings may help clinicians shorten the withdrawal time before the ^131^I treatment, which may reduce unnecessary impairments of stimulation to the foci from high TSH. Furthermore, using TSH to correct the predictive value of the sTg demonstrated that sTg/TSH ratio can be used as another predictor of ^131^I therapeutic effects. Considering the limitations of this study, follow-up research is looking forward to.

## Data Availability Statement

The raw data supporting the conclusions of this article will be made available by the authors, without undue reservation.

## Ethics Statement

The studies involving human participants were reviewed and approved by Ethics Committee of the Tianjin Medical University General Hospital. The patients/participants provided their written informed consent to participate in this study.

## Author Contributions

All authors listed have made a substantial, direct, and intellectual contribution to the work, and approved it for publication.

## Funding

Wei Zheng has a National Natural Science Foundation of China (No. 81601523) and a Health Science and Technology Project of Tianjin (ZC20181).

## Conflict of Interest

The authors declare that the research was conducted in the absence of any commercial or financial relationships that could be construed as a potential conflict of interest.
